# Three Active Phytotoxic Compounds from the Leaves of *Albizia richardiana* (Voigt.) King and Prain for the Development of Bioherbicides to Control Weeds

**DOI:** 10.3390/cells10092385

**Published:** 2021-09-10

**Authors:** Kawsar Hossen, Kaori Ozaki, Toshiaki Teruya, Hisashi Kato-Noguchi

**Affiliations:** 1 Department of Applied Biological Science, Faculty of Agriculture, Kagawa University, Miki 761-0795, Japan; kawsar.ag@nstu.edu.bd; 2 The United Graduate School of Agricultural Sciences, Ehime University, Matsuyama 790-8566, Japan; 3 Graduate School of Engineering and Science, University of the Ryukyus, 1 Senbaru, Okinawa 903-0213, Japan; kaorrira@gmail.com; 4 Faculty of Education, University of the Ryukyus, 1 Senbaru, Okinawa 903-0213, Japan; t-teruya@edu.u-ryukyu.ac.jp

**Keywords:** *Albizia richardiana*, phytotoxicity, biological management of weeds, compound **1** (4,5-dihydrovomifoliol), compound **2** (3-hydroxy-5α,6α-epoxy-β-ionone), compound **3** (3-(2-hydroxyethyl)-2,4,4-trimethyl-2cyclohexen-1-one)

## Abstract

The global population is increasing day by day. To meet the food demand for such a huge number of people, crop production must increase without damaging the environment, and to prevent synthetic chemical herbicides from polluting the environment, controlling weeds using bioherbicides is essential. Accordingly, using phytotoxic substances obtained from plants for biological weed management has attracted attention. The plant *Albizia richardiana* possesses phytotoxic compounds that have been previously recorded. Hence, we have conducted this research to characterize more phytotoxic compounds in *Albizia richardiana*. Aqueous methanolic extracts of *Albizia richardiana* plant significantly restricted the growth of the examined plants lettuce and Italian ryegrass in a species- and concentration-dependent manner. Three active phytotoxic compounds were isolated through various chromatographic methods and identified as compound **1**, **2**, and **3**. Compound **3** exhibited stronger phytotoxic potentials than the other two compounds and significantly suppressed the growth of *Lepidium sativum* (cress). The concentration of the compounds required for 50% growth reduction (I_50_ value) of the *Lepidium sativum* seedlings ranged between 0.0827 to 0.4133 mg/mL. The results suggest that these three phytotoxic compounds might contribute to the allelopathic potential of *Albizia richardiana*.

## 1. Introduction

By the year 2050, the global population might reach 9.2 billion, an increase of 30% compared with the present population. Due to the continuous increase in the world population, demand for food is also increasing. To feed such a huge number of people, crop production needs to increase 80% [1]. From the mid-nineteenth century (Green Revolution), crop yields have increased as a result of major developments in agriculture through the use of chemical fertilizers, irrigation, pesticides, synthetic herbicides and modern varieties of different crops. Although these technological developments have increased the production of food, they have also been responsible for the degradation of land, destruction of habitat, and depletion of the environment [2]. With the increasing of food production or crop production, pressure from weeds, insects, and diseases has also increased. Weeds have been considered dangerous crop pests from the earliest times of growing crops [3], suppressing production throughout the world. Weeds are a principal factor in influencing crop production worldwide because they compete with the crop plants for limited resources [4]. When weeds grow contemporaneously with crops, they decrease the productivity of those crops or reduce the quality of the harvested product because of competition for sunlight, water, space, and nutrients [5]. Weeds are regarded as a major pest, which is responsible for 45% of crop yield losses, while 25, 20, 15, and 6% of yield losses are caused by pathogens, insects, storage pests, and rodents, respectively [6]. Bajwa et al. [7] also reported that in the absence of weed control methods, yield losses exceed 70%. Accordingly, crop growers should devote a portion of production cost to the management of weeds in their crop fields. To reduce the disruptive effect of weeds in crops fields, applying appropriate weed control practices is essential [8]. The cost of these weed control practices to farmers varies between 5 and 20% of the whole production cost for different foods or crops [9], but in the case of field crops, the cost is about one third of the total [10].

To decrease crop yield losses caused by pests, and specifically weeds, control strategies are necessary. Throughout the history of land cultivation by humans, farmers or producers have attempted to control weeds to increase production, and as a result, weed management practices have evolved in tandem with scientific and technological advancements. Weeding by hand and using mechanical, cultural, physical, chemical, biological, and combined methods of controlling weeds are the basic weed control practices executed by farmers to reduce the deleterious effects of weeds on the agricultural farming systems. Despite all of the problems and issues associated with the use of synthetic herbicides, chemical control is still regarded as the most common and successful weed control method throughout the globe. A wide range of agrochemicals (insecticides, herbicides, nematicides, fungicides, etc.) is being used to control weeds and other crop pests in agriculture [11]. For instance, among various types of herbicides, glyphosate has been one of the most common nontarget, post-emergence herbicides applied in the agriculture sector for controlling weeds since 1970 [12]. Glyphosate herbicides have been selected for their strong activity against several species of weed plants [13]. After herbicides are applied in the crop fields, they can move along different pathways within the soil and the surrounding environment. Synthetic chemicals have severe morphological and physiological effects on weeds, such as leaf cupping, stunted growth, delayed flowering, signs of burning, necrosis, and malformed flowers [14]. For these reasons, herbicides are commonly used in agricultural farms, and they are also a cost-effective and realistic way to combat weeds.

However, herbicides persist in the soil atmosphere and can remain in the environment for long periods of time, posing a risk of soil contamination [15]. The extensive use of most herbicides in agriculture is a major concern among the public due to their harmful effects on the surrounding environment and on public health. On the other hand, presently, 502 distinct cases (species of weed × site of action), among them 263 species of weeds (dicots 152 and monocots 111), 21 of 31 specific herbicides’ sites of action and 164 types of herbicides, have developed resistance throughout the world. Herbicide resistant weeds have also been reported in 95 species of crops in 71 countries [16]. Reports on the direct harmful effects of chemical herbicides on crops have been published by many scientists in various regions and under different conditions. Depending on the chemical composition of the herbicide, condition of the environment, properties of the soil, and plant species, the negative effects of herbicide residues can be classified like total damage to crops, reduced biomass of the plants, and huge growth of crops. Moreover, production and germination of seeds, flowering, and plant viability can be influenced by the residue of herbicides during critical growth stages [17]. The difficulties with traditional weed management methods (weeding by hand, mechanical means, herbicides, etc.) necessitate the development of diverse weed management methods. If different weed control approaches can be developed, there will be a range of options for site-specific weed management. Using a variety of weed control solutions could effectively address cost and environmental issues. Using the phenomenon of allelopathy to suppress weeds is one of the most effective weed management methods [18].

Allelopathy is a natural phenomenon in which secondary metabolites or allelochemicals are produced by one organism. These allelochemicals affect the germination, development, reproduction, and survival of other organisms in the same population [19]. In an agro-ecosystem, organisms, including plants, cannot live together, without having beneficial or harmful effects on one another. These effects are because of the allelochemicals released by nearby plants [20]. Allelopathy reduces and disrupts the germination of crop seeds and restricts the growth of shoots and roots [21]. Most of the plant physiological mechanisms such as photosynthesis, functions of membranes and enzymes, water and nutrient uptake and germination of seedlings are influenced by allelochemicals [22,23]. Moreover, phytochemicals may be applied as bioherbicides to increase crop production by means of biological weed management through allelopathy. These substances may also aid people’s health by reducing the risk of mutagenic, genotoxic, and cytotoxic effects [24]. One of the key effects of allelopathy is phytotoxicity, which can occur naturally or as a result of using phytochemicals as bioherbicides [25]. Due to their destructive effect on plants (growth and development), various allelopathic compounds like sterols and terpenes [26], fatty acids [27], and phenolic substances [28] are examples of natural substances applied to control weeds in organic agricultural systems [29]. Herbicides based on natural compounds have comparatively short half-lives and process relatively few halogen groups, indicating that bioherbicides degrade quickly, and residues do not remain in the soil following harvesting of crops [30]. Hence, secondary metabolites from plant or natural sources can be used to control weeds, which helps to protect the environment as well as people.

*Albizia richardiana* (Voigt.) King and Prain plant species (Bangladesh name: chambal or rajkoroi) are a fast growing, very big, and deciduous plants of the Fabaceae family [31]. The leaves of *Albizia richardiana* are sessile, bipinnate, small, and compound. The tree is white tinged with green, small, and has stalkless flowers. It also bears white-brown, long, and thin fruits. This plant species is generally found in warm, humid regions in Africa, Madagascar, Asia, North America, and Australia, but is most common in the Old World [32]. In Bangladesh, the plant grows in various regions like Barisal, Sunamgonj, Madaripur, Chittagong, Pirujpur, Jhalukati and Bagerhat. This species is planted for ornamental purposes, and is also grown to enhance the attractiveness of roadsides due to its beautiful structure [33]. *Albizia richardiana* plays a vital role in community forest development of Bangladesh as well [34]. The wood of the tree is usually used to make different types of furniture, posts, frames, roofing materials, and plywood [35]. Various parts like the bark, roots, flowers, and fruits have many pharmaceutical properties that are used to treat different maladies like loss of appetite, depression, chest tightness, eye problems, blurred vision, and back pain, and to help maintain blood flow in the body [36]. Different compounds such as alkaloids, glycosides, saponins, glucosides, and carbohydrates are found in the bark of the plant. Anti-inflammatory, hypoglycemic, and antimicrobial effects have also been reported [37].

Although various studies have been conducted on the different pharmaceutical potentials and other uses of *Albizia richardiana*, there is very little information in the literature about its phytotoxicity. Hence, this study was conducted to explore the allelopathic potential of, and to find more phytotoxic compounds that may be helpful for developing of bioherbicides in, *Albizia richardiana*.

## 2. Materials and Methods

### 2.1. General Experimental Procedures

All NMR data were recorded on a Bruker Avance III 500 (Bruker Corporation, Billerica, MA, USA) for ^1^H (500 MHz) and ^13^C (125 MHz). Chemical shifts were reported relative to the residual solvent signals (CDCl_3_: δ_H_ 7.26, δ_C_ 77.2, CD_3_OD: δ_H_ 3.31). HRESIMS data were obtained using a Waters Micromass Q-TOF (Waters Corporation, Milford, MA, USA). HPLC was carried out with a JASCO PU-2080 Plus Intelligent HPLC pump and a JASCO UV-2075 Plus Intelligent UV/VIS detector.

### 2.2. Plant Samples

The leaves of *Albizia richardiana* plants were collected from different areas of Bangladesh Agricultural University (BAU), Mymensigh, Bangladesh, in the middle of 2019 (June to August). The collected leaves were washed under running tap water. The washed leaves were air-dried in a shady place, and then ground into powder using a grinder machine. Last, the leaf powder was deposited in a polyethene bag and kept in a refrigerator at 2 °C until extract preparation. For the phytotoxic growth potential experiments, lettuce (*Lactuca sativa* L.), and Italian ryegrass (*Lolium multiflorum* Lam.) were used as examine plant species. Lettuce is a dicotyledonous plant and its growth patterns are known, whereas the Italian ryegrass is considered a familiar weed in crop fields.

### 2.3. Extraction and Growth Assay with the Albizia Richardiana Extracts

The powder (100 g) made from the leaves of *Albizia richardiana* was mixed with 500 mL of 70% (*v*/*v*) aqueous methanol for 48 h. The leaf extracts were filtrated using a layer of filter paper (No. 2, 125 mm; Advantec, Toyo Roshi Kaisha Ltd., Tokyo, Japan), and the residue mixture was re-extracted with 500 mL of methanol for 24 h then filtrated again. These two residues of leaf powder were mixed and evaporated with a rotary evaporator at 40 °C. The leaf extracts were dissolved in methanol (250 mL) to produce bioassay concentrations of 3, 10, 30, 100, 300, and 1000 mg dry weight (DW) equivalent extract/mL, and assay tests were conducted with the *Albizia richardiana* crude extracts against the selected species for this study as recommended by Hossen et al. [38]. The exact quantity of residues was transferred to a filter paper (No. 2, 28 mm; Toyo Roshi Ltd., Tokyo, Japan) on 28 mm Petri dishes. The Petri dishes containing the methanolic residues were then desiccated in a draft chamber, and 0.6 mL of 0.05% (*v*/*v*) aqueous solution of Tween 20 (polyoxyethylene sorbitan monolaurate; Nacalai Tesque, Inc., Kyoto, Japan) (acts as a surface active agent without any harmful effect) was added to each dish. The seeds or germinated seeds of chosen plants were then placed onto the filter paper in the Petri dishes. Only Tween 20 solution was applied for the control experiments.

### 2.4. Purification of the Active Compounds

**Figure 1:**
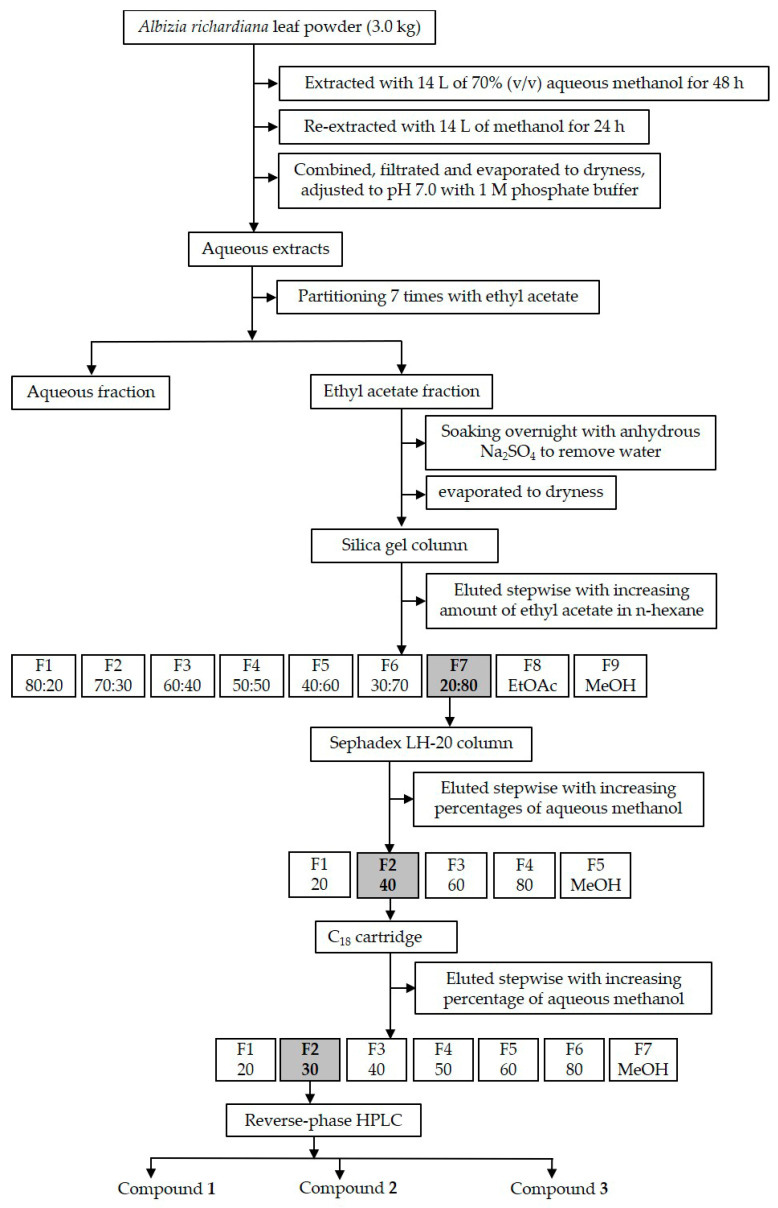
Extraction procedure and purification of compound **1**, **2**, and **3** (4,5-dihydrovomifoliol, 3-hydroxy-5α,6α-epoxy-β-ionone, and 3-(2-hydroxyethyl)-2,4,4-trimethyl-2cyclohexen-1-one).

The leaves of *Albizia richardiana* (3.0 kg) were extracted as described above. The obtained leaf extracts were then dried using a rotary evaporator (40 °C) to produce H_2_O (aqueous) residues, and the pH was adjusted to 7.0 by adding 1.0 M phosphate-buffered solution. The H_2_O residues were partitioned seven times with an equal amount of EtOAc (ethyl acetate) and segregated into H_2_O and EtOAc fractions. To remove H_2_O from the EtOAc fraction, anhydrous Na_2_SO_4_ was used. With the H_2_O and EtOAc fractions, an assay experiment was conducted with *Lepidium sativum* (cress). The EtOAc fraction had higher inhibitory effects compared with the H_2_O fraction (data not shown). Hence, the EtOAc fraction was selected for subsequent bioassay-guided fractionations through several purification steps: a column of silica gel, column of Sephadex LH-20, C_18_ cartridge, and reverse-phase HPLC (500 × 10 mm I.D. ODS AQ-325; YMC Ltd., Kyoto, Japan). Phytotoxic activity was measured using the *Lepidium sativum* (cress) assay experiment for every chromatographic step according to Hossen et al. [38], resulting in the isolation of three compounds: compound **1**, **2**, and **3** (4,5-dihydrovomifoliol, 3-hydroxy-5α,6α-epoxy-β-ionone, and 3-(2-hydroxyethyl)-2,4,4-trimethyl-2cyclohexen-1-one) (Figure 1). These compounds were then purified once again using reverse-phase HPLC (4.6 × 250 mm I.D., S-5 µm, Inertsil ^®^ ODS-3; GL Science Inc., Tokyo, Japan) at a flow rate 0.8 mL/min with 25% aqueous methanol obtained at retention times of 72–78, 80–84, and 90–102 min. Lastly, these three compounds were characterized through spectral analysis.

### 2.5. Growth Assay of the Characterized Compounds

The characterized compounds were mixed with 4 mL of MeOH (methanol) to prepare various bioassay concentrations of 0.00678, 0.0226, 0.0678, 0.226, 0.339, and 0.452 mg/mL for compound **1**, 0.00672, 0.0224, 0.0672, 0.224, 0.336, and 0.448 mg/mL for compound **2**, and 0.00547, 0.0183, 0.0547, 0.183, 0.274, and 0.365 mg/mL for compound **3**. The *Lepidium sativum* seeds were treated with these assay concentrations to check the growth activity as mentioned above (Section 2.3).

### 2.6. Statistics

The bioassay experiments were conducted using a completely randomized block design (CRBD) with three replications, and all the replications were repeated two times. The results are presented as mean ± standard error (SE). Analysis of variance (ANOVA) was determined by using SPSS statistical software, version 20.0 for Windows (SPSS Inc., Chicago, IL, USA). Significant differences (between the control treatment and sample treatments) were determined using Tukey’s honestly significant difference (HSD) test at the 0.05 level of significance [39]. The I_50_ values (concentrations required for 50% suppression of the growth of the test plants) was calculated using GraphPad Prism ^®^Ver. 6.0 (GraphPad Software, Inc., La Jolla, CA, USA).

## 3. Results

### 3.1. Phytotoxic Effect of the Albizia Richardiana Extracts

**Figure 2:**
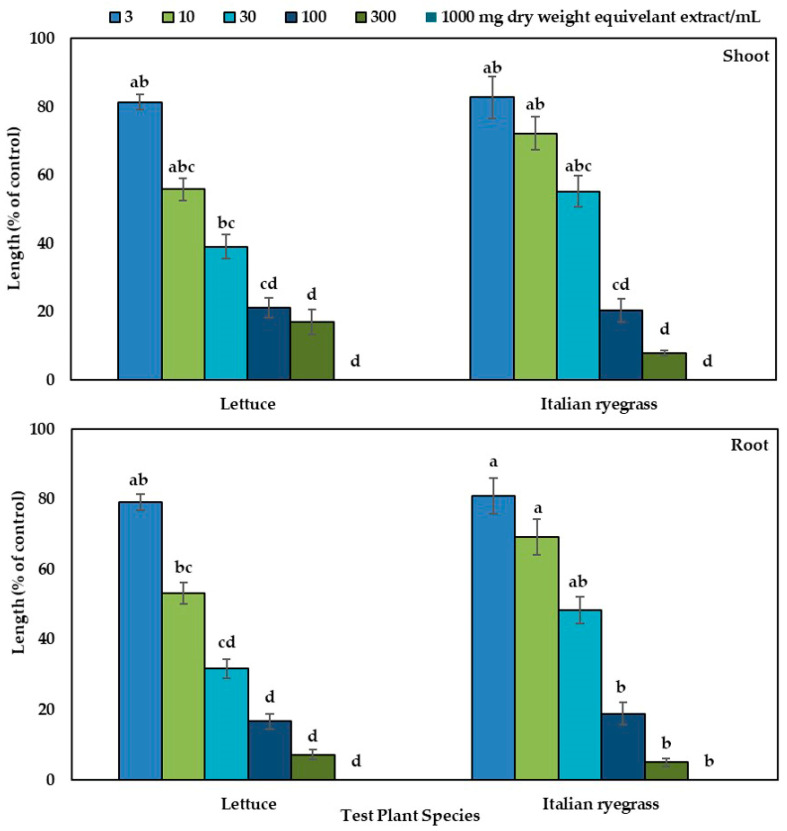
Phytotoxicity of the H_2_O methanolic extracts of *Albizia richardiana* on the growth of the shoots and roots of the seedlings of *Lactuca sativa* (lettuce) and *Lolium multiflorum* (Italian ryegrass) at different concentrations. Mean ± SE (standard error) was calculated from two separate studies (replicated three times; number of seedlings /treatments was 10, and total seedlings *n* = 60). Different letters indicate significant variations according to homogenous subsets of Tukey’s HSD test at the 0.05 level of significance.

The phytotoxicity of the H_2_O (aqueous) methanolic extracts of the *Albizia richardiana* plant are exhibited in Figure 2, and suppression increased with increasing extract concentrations. Significant growth restriction started from a concentration of 10 mg DW (dry weight) equivalent extract/mL, except for the root growth of *Lolium multiflorum* (Italian ryegrass). At a concentration 300 mg DW (dry weight) equivalent extract/mL, shoot and root growth of the *Lactuca sativa* (lettuce) was inhibited to 17.0 and 7.22%, respectively, of control, while the growth of *Lolium multiflorum* was inhibited to 2.82 and 5.03%, respectively. The *Lactuca sativa* and *Lolium multiflorum* seeds did not germinate or were completely inhibited at a concentration of 1000 mg DW equivalent extract/mL. The I_50_ values of the *Albizia richardiana* extracts for the examined plants ranged from 11.0 to 32.0 mg DW equivalent extract/mL as shown in Table 1.

### 3.2. Identification of Active Phytotoxic Compounds

**Figure 3:**
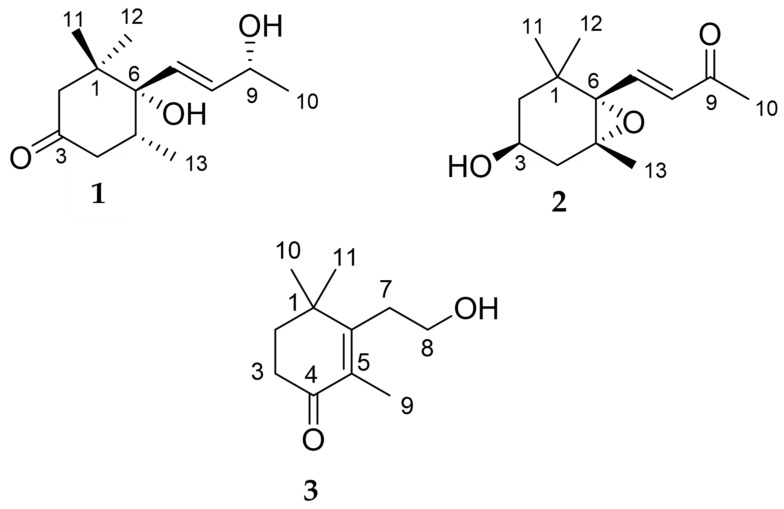
The molecular structures of the identified phytotoxic compounds **1**, **2**, and **3** (4,5-dihydrovomifoliol, 3-hydroxy-5α,6α-epoxy-β-ionone, and 3-(2-hydroxyethyl)-2,4,4-trimethyl-2cyclohexen-1-one) from the *Albizia richardiana* extract.

The molecular formula of compound **1** was found to be C_13_H_22_O_3_ through HRESIMS (Figure S8). The ^1^H NMR spectrum of compound **1** (Figure S1) as measured in CD_3_OD showed the presence of four methyl proton signals at δ_H_ 1.27 (3H, d, J = 6.4), 0.98 (3H, s), 0.92 (3H, s), and 0.90 (3H, d, J = 6.7), two olefinic proton signals at δ_H_ 5.84 (1H, dd, J = 15.7, 5.8), and 5.66 (1H, dd, J = 15.7, 1.2), four methylene proton signals at δ_H_ 2.87 (1H, d, J = 13.6), 2.45 (1H, dd, J = 14.1, 12.1), 2.12 (1H, ddd, J = 14.1, 4.6, 2.3), and 1.82 (1H, dd, J = 13.6, 2.3), and two methine proton signals at δ_H_ 4.34 (1H, q), and 2.27 (1H, m). The ^1^H NMR spectrum of compound **1** was in agreement with previously reported data leading to the identification of this compound as 4,5-dihydrovomifoliol (Figure 3) [40].

The molecular formula of compound **2** was found to be C_13_H_20_O_3_ by HRESIMS (Figure S9). The ^1^H NMR spectrum of compound **2** (Figure S2) as measured in CD_3_OD showed the presence of four methyl proton signals at δ_H_ 2.29 (3H, s), 1.19 (3H, s), 1.18 (3H, s), and 0.96 (3H, s), two olefinic proton signals at δ_H_ 7.17 (1H, d, J = 15.8) and 6.18 (1H, d, J = 15.8), four methylene proton signals at δ_H_ 2.30 (1H, ddd, J = 14.3, 5.1, 1.7), 1.66 (1H, dd, J = 14.3, 9.2), 1.58 (1H, ddd, J = 12.9, 3.4, 1.7), and 1.27 (1H, dd, J = 12.9, 10.8), and one methine proton signal at δ_H_ 3.76 (1H, m). The ^1^H NMR spectrum of compound **2** was in agreement with previously reported data leading to the identification of this compound as 3-hydroxy-5α,6α-epoxy-β-ionone [(3S, 5R, 6S, 7E)-5,6-epoxy-3-hydroxy-7-megastigmen-9-one] (Figure 3) [41].

**Figure 4:**
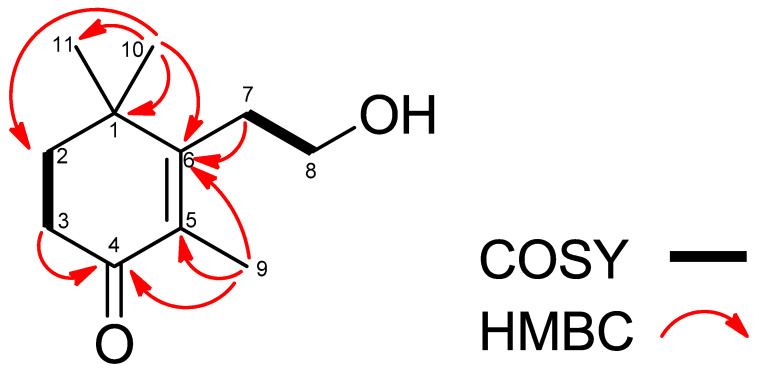
Gross structure of compound **3** or 3-(2-hydroxyethyl)-2,4,4-trimethyl-2cyclohexen-1-one as determined by 2D-NMR spectroscopy.

Compound **3** was obtained as a colorless oil. The molecular formula of compound **3** was found to be C_11_H_18_O_2_ by HRESIMS (Figure S10). The ^1^H NMR spectrum of compound **3** (Figure S3) as measured in CDCl_3_ showed two methyl proton signals at δ_H_ 1.81 (3H, s) and 1.17 (6H, s), and four methylene proton signals at δ_H_ 3.73 (2H, t, J = 8.0), 2.58 (2H, t, J = 8.0), 2.47 (2H, t, J = 6.6), and 1.81 (2H, t, J = 6.6). The ^13^C NMR spectrum (Figure S4) showed one ketone carbon signal at δ_C_ 198.9, and two quaternary carbon signals at δ_C_ 160.0 and 132.5. The gross structure was determined on the basis of 1D and 2D NMR experiments. A detailed analysis of the COSY (Figure S7) and HSQC spectra (Figure S5) of compound **3** revealed the two partial structures C-2 to C-3 and C-7 to C-8. In the HMBC spectrum (Figure S6), H-9 showed correlations with to C-1, C-2, C-6, and C-11, H-3 showed a correlation with C-4, and H-7 showed a correlation with C-6. Thus, the gross structure of compound **3** was determined as 3-(2-hydroxyethyl)-2,4,4-trimethyl-2cyclohexen-1-one, shown in Figure 4, and one methine proton signal at δ_H_ 3.76 (1H, m). The ^1^H NMR spectrum of compound **3** was in agreement with previously reported data leading to the identification of this compound as 3-(2-hydroxyethyl)-2,4,4-trimethyl-2cyclohexen-1-one (Figure 3) [42].

### 3.3. Inhibitory Effects of the Allelopathic Compounds

**Figure 5:**
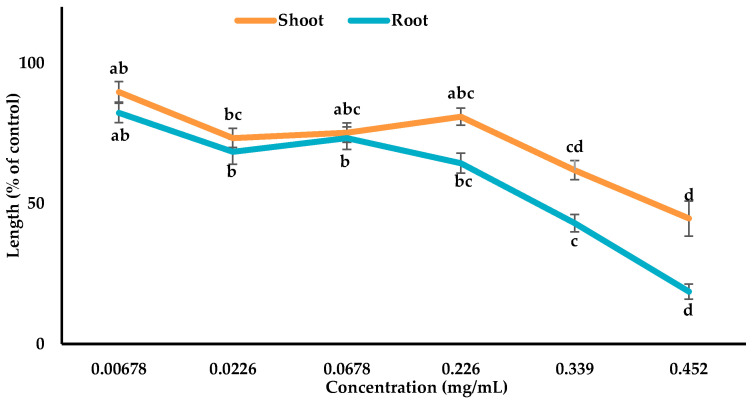
Phytotoxic potential of compound **1** against the shoot and root growth of *Lepidium sativum*. The values are the mean ± standard error (SE) from two different studies with 10 seedlings for each treatment. Different letters indicate significant variations according to Tukey’s HSD test at the 0.05 level of significance.

**Figure 6:**
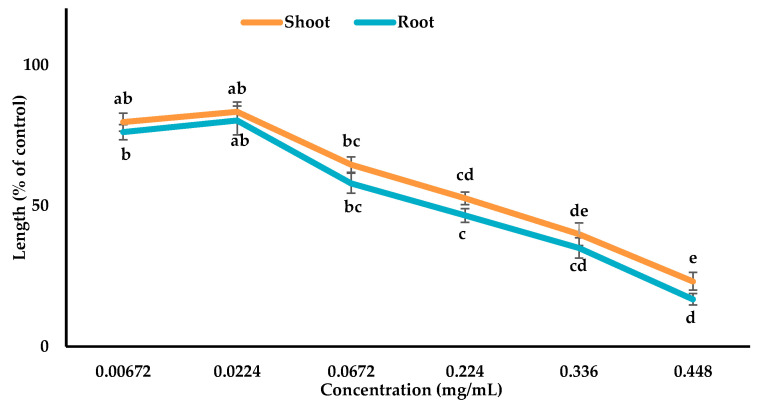
Phytotoxic potentials of compound **2** against the shoot and root growth of *Lepidium sativum*. The values are the mean ± standard error (SE) from two different studies with 10 seedlings for each treatment. Different letters indicate significant variations according to Tukey’s HSD test at the 0.05 level of significance.

**Figure 7:**
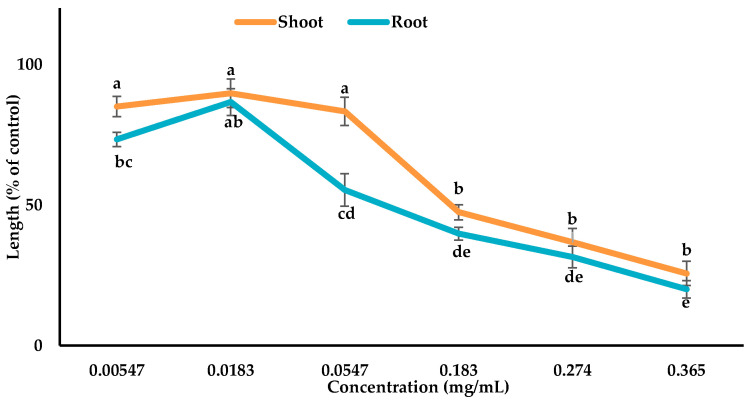
Phytotoxic potentials of compound **3** against the shoot and root growth of *Lepidium sativum*. The values are the mean ± standard error (SE) from two different studies with 10 seedlings for each treatment. Different letters indicate significant variations according to Tukey’s HSD test at the 0.05 level of significance.

The phytotoxic potential of compounds **1**, **2**, and **3** isolated from *Albizia richardiana* was checked using the growth assay of *Lepidium sativum* and the growth suppression was dependent on the concentrations (Figure 5, Figure 6 and Figure 7). The *Lepidium sativum* seedlings displayed the maximum restriction (shoot 55.35% and root 81.4%) with compound **1**, (shoot 76.9% and root 83.25%) with compound **2**, and (shoot 74.36% and root 80.00%) with compound **3** at the maximum concentration. The I_50_ values for the *Lepidium sativum* seedlings varied from 0.2845 to 0.4133 mg/mL for compound **1**, 0.1552 to 0.2432 mg/mL for compound **2**, and 0.0827 to 0.1671 mg/mL for compound **3** (Table 2). From the I_50_ value, it was observed that compound **3** showed more allelopathic activity than the other two compounds (Table 2).

## 4. Discussion

In our two previous studies, we examined the phytotoxicity of extracts of *Albizia richardiana* leaves against the seedling growth of *Lepidium sativum*, *Medicago sativa* L. (crop plants), *Echinochloa crus-galli* (L.) P. *Beauv.* and *Phleum pratense* L. (species of weeds) and observed notable growth suppression [38,43]. To validate those results, in this study, we also examined the allelopathic potential of the extracts of *Albizia richardiana* leaves against the growth of *Lactuca sativa* (lettuce) and *Lolium multiflorum* (Italian ryegrass). Aqueous methanolic extracts of *Albizia richardiana* leaves significantly limited the growth (shoot and root) of the tested plant species, and the level of suppression increased with increasing extract concentration. Other studies have also reported the same types of concentration-dependent suppression using extracts of various plant species [44,45,46,47]. The I_50_ values of the *Albizia richardiana* leaf extracts indicated that growth suppression was tested species dependent (Table 1). In allelopathic research, sensitivity to extracts varies with different target plant species [48,49,50,51], suggesting variations in absorption mechanism, translocation, and sites of action of the phytochemicals across plant species [52]. Imatomi et al. [49] reported that the effects of plant extract on the germination of seeds depend on different seed attributes such as size, shape, structure, and seed coat permeability. The plant species-dependent activity of phytochemicals shows that these phytochemicals are essential not only for the environment and floristic compositions, but also for the agricultural farming systems, where allelochemicals can be applied as bioherbicides. The growth inhibitory potential of the extracts from *Albizia richardiana* leaves suggest that this plant species might possess allelopathic compounds.

Phytotoxic compounds can affect various plant physiological processes of plants like germination of seeds, uptake of ions, photosynthesis, opening of stromata, respiration, water status, transpiration, activity of enzymes, and hormone conditions [53]. Phytotoxic compounds also affect the cell division and elongation, expression of genes, transduction of signals, structure of cell walls and membranes, and the permeability of cells [54,55]. The results of the present study show that the shoot growth of the tested plants was less sensitive to the *Albizia richardiana* leaf extracts than the root growth. The higher susceptibility of the roots compared with shoots is common, because the roots of plants are the first plant organ that absorb allelopathic compounds from extracts, and root tissues are more permeable than shoot tissues [56,57], and Franco et al. [58] reported that phytochemicals can influence the genes responsible for the cellular depiction of underground endoderm and tissues, decreasing root development. Levizou et al. [59] reported that less mitotic cell division in the apex of roots results in more root growth suppression of lettuce (*Lactuca sativa*) when exposed to extracts of *Dittrichia viscose* leaves. Such kinds of suppression have also been reported in many other studies [58,60,61,62,63]. Measuring the lengthening or shortening of shoots and roots is commonly employed to determine allelopathic activity [64]. In our two previous studies, two phytochemical substances, dehydrovomifoliol and loliolide [38], and another two allelopathic substances, 3-hydroxy-4-oxo-β-dehydroionol (novel compound) and 3-oxo-α-ionone [43], were isolated and identified from *Albizia richardiana* leaf extracts. In this study, we have also isolated another three phytochemical substances from *Albizia richardiana* leaf extracts: compounds **1**, **2**, and **3** (Figure 3).

Compound **1** is a volatile C_13_-norisoprenoidic compound released through glycoside enzyme hydrolysis from *Vitis vinifera* (Melon B. grapes), and involved in the development of aroma in grapes [65]. This compound has also been identified in different plants such as *Prunus persica* [66], different varieties of grapes [67], and starfruits [68]. Although compound **1** has been isolated from many plants, there have been no reports in the literature about its phytotoxicity. This study is the first to document the identification of compound **1** from the leaf extracts of *Albizia richardiana* as well as its phytotoxic potential. Compound **2** is a norisoprenoid derivatives, first identified by Kim et al. [41] from the upper parts of chard (*Beta vulgaris* var. *cicla*), it has also been isolated from other plants like *Murraya koenigii* [69], red beetroot [70], and *Herpetospermum pedunculosum* [71]. Although compound **2** and its medicinal properties have already been reported, there has been no evidence of its allelopathic potential. This study is also the first report on the isolation of this compound from *A. richardiana* leaf extracts and its allelopathic properties. The compound **3** is a homo-monoterpene first isolated by Takikawa et al. [42]. This report is the first to document the identification of compound **3** from leaf extracts of *Albizia richardiana* and its phytotoxicity.

The results obtained from the current research indicate that compound **1**, **2**, and **3** significantly restricted the seedling growth of *Lepidium sativum* (Figure 5, Figure 6 and Figure 7). The I_50_ values showed that compound **3** exhibited stronger phytotoxic potential than the other two compounds (Table 2). The variations in the phytotoxic effects of the compounds might result from the differences in their molecular structures, because the phytotoxicity of allelopathic compounds is determined by their structural variations [72,73].

Therefore, based on the of the above discussion, compound **1**, **2**, and **3** are responsible for the phytotoxicity of the *Albizia richardiana* leaves. Accordingly, the phytotoxic potentials of the *Albizia richardiana* plant leaves might be helpful for reducing the application of synthetic chemical herbicides and also to avoid the harmful effects of these herbicides on the environment and human health.

## 5. Conclusions

The H_2_O (aqueous) methanolic extracts of *Albizia richardiana* leaves limited the growth (shoot and root) of examined plant species such as *Lactuca sativa* and *Lolium multiflorum*, and the level of growth suppression were varied with the extract concentration and examined plant species. Three phytotoxic compounds were isolated from the *Albizia richardiana* leaves through different chromatographic steps and identified through spectroscopic analysis as compound **1**, **2**, and **3**. All three substances significantly limited the growth of *Lepidium sativum* seedlings. The results of this study suggest that the three compounds are responsible for the phytotoxicity of *Albizia richardiana*. Therefore, this plant species may be able to play a vital role in the biological control of weeds.

## Figures and Tables

**Table 1:** The I_50_ values (concentrations resulting in 50% growth restriction) of the *Albizia richardiana* H_2_O (aqueous) methanolic extracts against the test plants.

Tested Species		I_50_ Values (mg Dry Weight Equivalent Extract/mL)	
		Shoot	Root
Dicot	*Lactuca sativa* (lettuce)	14.0	11.0
Monocot	*Lolium multiflorum* (Italian ryegrass)	32.0	28.0

**Table 2:** The I_50_ values (concentrations resulting in 50% growth restriction) of compound **1**, **2**, and **3** against the test plant.

Test Plant		Compound 1	Compound 2	Compound 3
		(mg/mL)		
*Lepidium sativum* (Cress)	Shoot	0.4133	0.2432	0.1671
	Root	0.2845	0.1552	0.0827

## Data Availability

Not applicable.

## Supplementary Materials

The following are available online at https://www.mdpi.com/article/10.3390/cells10092385/s1, NMR spectral data and mass spectral data of compound **1**, **2**, and **3** (4,5-dihydrovomifoliol, 3-Hydroxy-5α,6α-epoxy-β-ionone, and 3-(2-hydroxyethyl)-2,4,4-trimethyl-2cyclohexen-1-one) on page S2, Figure S1: ^1^H NMR spectrum of compound **1** (4,5-dihydrovomifoliol) in CD_3_OD (500 MHz), Figure S2: ^1^H NMR spectrum of compound **2** (3-Hydroxy-5α,6α-epoxy-β-ionone) in CD_3_OD (500 MHz), Figure S3: ^1^H NMR spectrum of compound **3** (3-(2-hydroxyethyl)-2,4,4-trimethyl-2cyclohexen-1-one) in CDCl_3_ (500 MHz), Figure S4: ^13^C NMR spectrum of compound **3** (3-(2-hydroxyethyl)-2,4,4-trimethyl-2cyclohexen-1-one) in CDC_l3_ (125 MHz), Figure S5: HSQC spectrum of compound **3** (3-(2-hydroxyethyl)-2,4,4-trimethyl-2cyclohexen-1-one) in CDC_l3_ (500 MHz), Figure S6: HMBC spectrum of compound **3** (3-(2-hydroxyethyl)-2,4,4-trimethyl-2cyclohexen-1-one) in CDC_l3_ (500 MHz), Figure S7: COSY spectrum of compound **3** (3-(2-hydroxyethyl)-2,4,4-trimethyl-2cyclohexen-1-one) in CDC_l3_ (500 MHz), Figure S8: HRESIMS spectrum of compound **1** (4,5-dihydrovomifoliol), Figure S9: HRESIMS spectrum of compound **2** (3-Hydroxy-5α,6α-epoxy-β-ionone), Figure S10: HRESIMS spectrum of compound **3** (3-(2-hydroxyethyl)-2,4,4-trimethyl-2cyclohexen-1-one).
