# Supplementary material for: Three Active Phytotoxic Compounds from the Leaves of Albizia richardiana (Voigt.) King and Prain for the Development of Bioherbicides to Control Weeds
Source: Cells. 2021 Sep 10;10(9):2385. doi: 10.3390/cells10092385 (PMC8472145; doi:10.3390/cells10092385)
Supplement: Supplementary file 1 [file cells-10-02385-s001.zip › cells-1284750-supplementary.pdf]

Supporting Information:

Three Active Phytotoxic Compounds from the Leaves of *Albizia richardiana* (Voigt.) King and Prain for the Development of Bioherbicides to Control Weeds

Kawsar Hossen [1,2], Kaori Ozaki [3], Toshiaki Teruya [4] and Hisashi Kato-Noguchi [1,2,*]

[1] Department of Applied Biological Science, Faculty of Agriculture, Kagawa University, Miki 761-0795, Japan; kawsar.ag@nstu.edu.bd
[2] The United Graduate School of Agricultural Sciences, Ehime University, Matsuyama 790-8566, Japan
[3] Graduate School of Engineering and Science, University of the Ryukyus, 1 Senbaru, Nishihara, Okinawa 903-0213, Japan; k168239@eve.u-ryukyu.ac.jp
[4] Faculty of Education, University of the Ryukyus, 1 Senbaru, Nishihara, Okinawa 903-0213, Japan; t-teruya@edu.u-ryukyu.ac.jp
* Correspondence: kato.hisashi@kagawa-u.ac.jp

Contents **Page Number**

NMR spectral data and Mass spectral data of compound 1, 2, 3 (4,5-dihydrovomifoliol, 3-Hydroxy-5α,6α-epoxy-β-ionone, and 3-(2-Hydroxyethyl)-2,4,4-trimethyl 2cyclohexen-1-one)	S2
NMR spectrum of compound 1, 2 (4,5-dihydrovomifoliol, and 3-Hydroxy-5α, 6α-epoxy-β-ionone)	S3
NMR spectrum of compound 3 (3-(2-Hydroxyethyl)-2,4,4-trimethyl-2cyclohexen-1-one)	S4
HRESIMS spectrum of compound 1, 2, 3 (4,5-dihydrovomifoliol, 3-Hydroxy-5α,6α- epoxy-β-ionone, and 3-(2-Hydroxyethyl)-2,4,4-trimethyl-2cyclohexen-1-one)	S7

4,5-dihydrovomifoliol: colorless oil; ^{1}H NMR (500 MHz, CD_3OD) 5.84 (1H, dd, *J* = 15.7, 5.8 Hz, H-8), 5.66 (1H, dd, *J* = 15.7, 1.2 Hz, H-7), 4.34, (1H, q, H-9), 2.87 (1H, d, *J* = 13.6 Hz, H-2 ax), 2.45 (1H, dd, *J* = 14.1, 12.1 Hz, H-4 ax), 2.31-2.22 (1H, m, H-5), 2.12 (1H, ddd, *J* = 14.1, 4.6, 2.3 Hz, H-4 eq), 1.82 (1H, dd, *J* = 13.6, 2.3, H-2 eq), 1.27 (3H, d, *J* = 6.4 Hz, H_3-13), 0.98 (3H, s, H_3-11), 0.92 (3H, s, H_3-12), 0.90 (3H, d, *J* = 6.7 Hz, H_3-13); HRESIMS *m/z* 249.1463 (calcd for $C_{13}H_{22}O_3Na$, 249.1467).

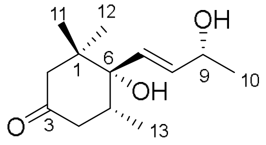

3-Hydroxy-5α,6α-epoxy-β-ionone; colorless oil; ^{1}H NMR (500 MHz, CD_3OD) 7.17 (1H, d, *J* = 15.8 Hz, H-7), 6.18 (1H, d, *J* = 15.8 Hz, H-8), 3.76 (1H, m, H-3), 2.30 (1H, ddd, *J* = 14.3, 5.1, 1.7 Hz, H-4), 2.29 (3H, s, H-10), 1.66 (1H, dd, *J* = 14.3, 9.2 Hz, H-4), 1.58 (1H, ddd, *J* = 12.9, 3.4, 1.7 Hz, H-2), 1.27 (1H, dd, *J* = 12.9, 10.8 Hz, H-2), 1.19 (3H, s, H-13), 1.18 (3H, s, H-12), 0.96 (3H, s, H-11); HRESIMS *m/z* 247.1306 (calcd for $C_{13}H_{20}O_3Na$ 247.1310).

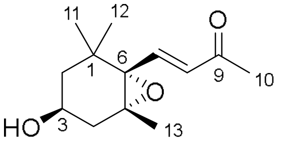

3-(2-Hydroxyethyl)-2,4,4-trimethyl-2cyclohexen-1-one; colorless oil; ^{1}H NMR (500 MHz, $CDCl_3$) 3.73 (2H, t, *J* = 8.0 Hz, 8-H), 2.58 (2H, t, *J* = 8.0 Hz, 7-H), 2.47 (2H, t, *J* = 6.6 Hz, 3-H), 1.81 (3H, s, 9-H), 1.81 (2H, t, *J* = 6.6 Hz, 2-H), 1.17 (6H, s, 11-H and 10-H); ^{13}C NMR (125 MHz, $CDCl_3$) 199.3, 160.0, 141.5, 132.5, 61.4, 37.4, 36.3, 34.4, 27.0, 12.0; HRESIMS *m/z* 183.1385 (calcd for $C_{11}H_{19}O_2$ 183.1385).

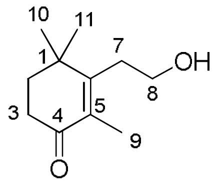

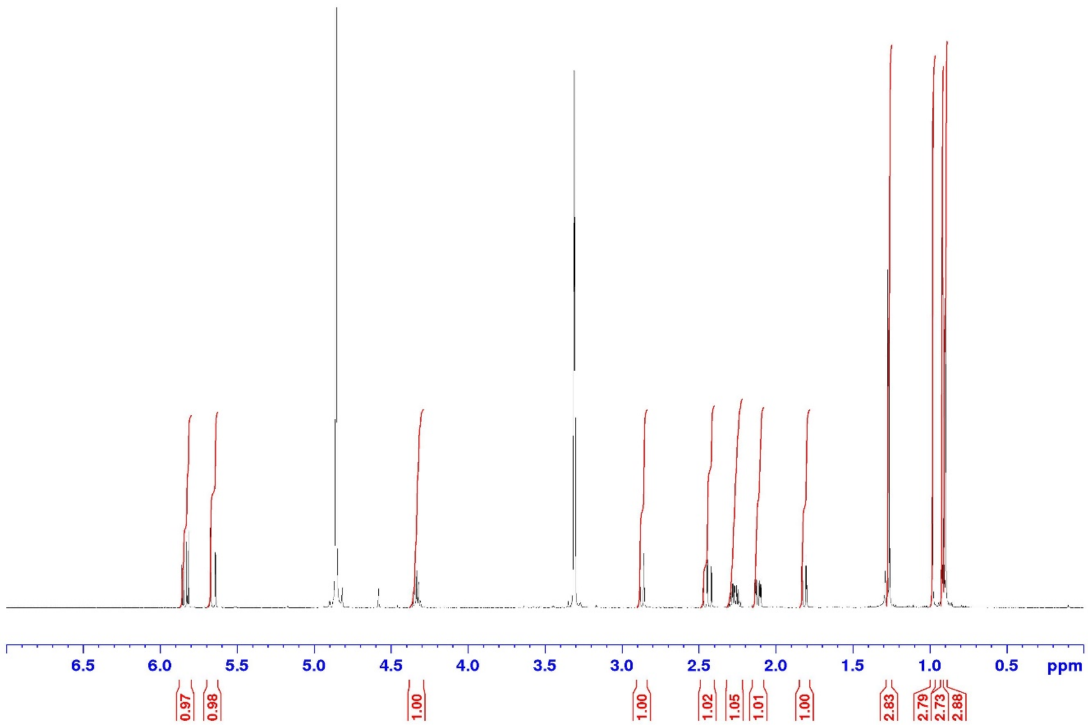

Figure S1. ^{1}H NMR spectrum of compound 1 (4,5-dihydrovomifoliol) in CD_3OD (500 MHz).

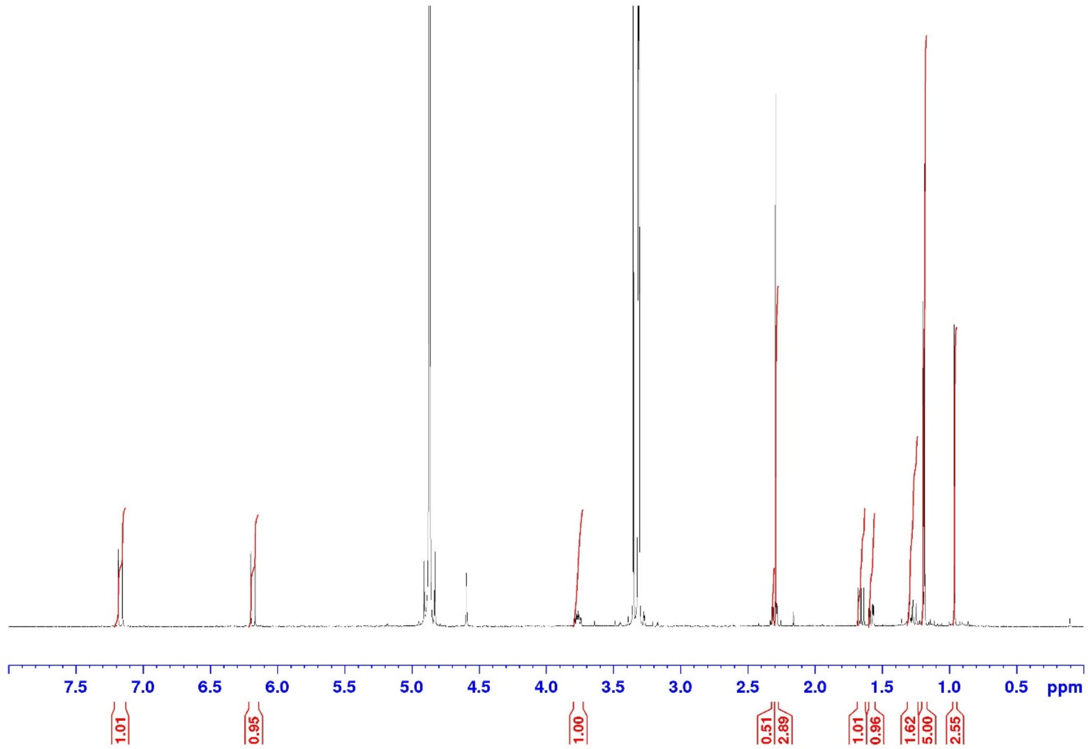

Figure S2. ^{1}H NMR spectrum of compound 2 (3-Hydroxy-5α,6α-epoxy-β-ionone) in CD_3OD (500 MHz).

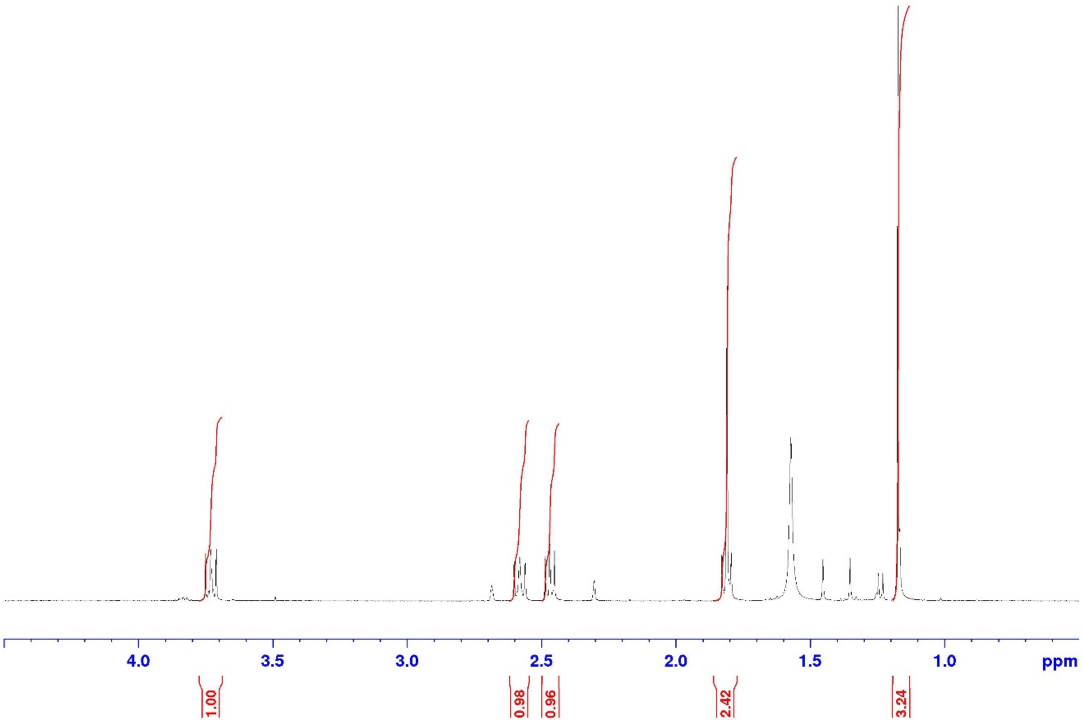

Figure S3. ^{1}H NMR spectrum of compound 3 (3-(2-Hydroxyethyl)-2,4,4-trimethyl-2cyclohexen-1-one) in $CDCl_3$ (500 MHz).

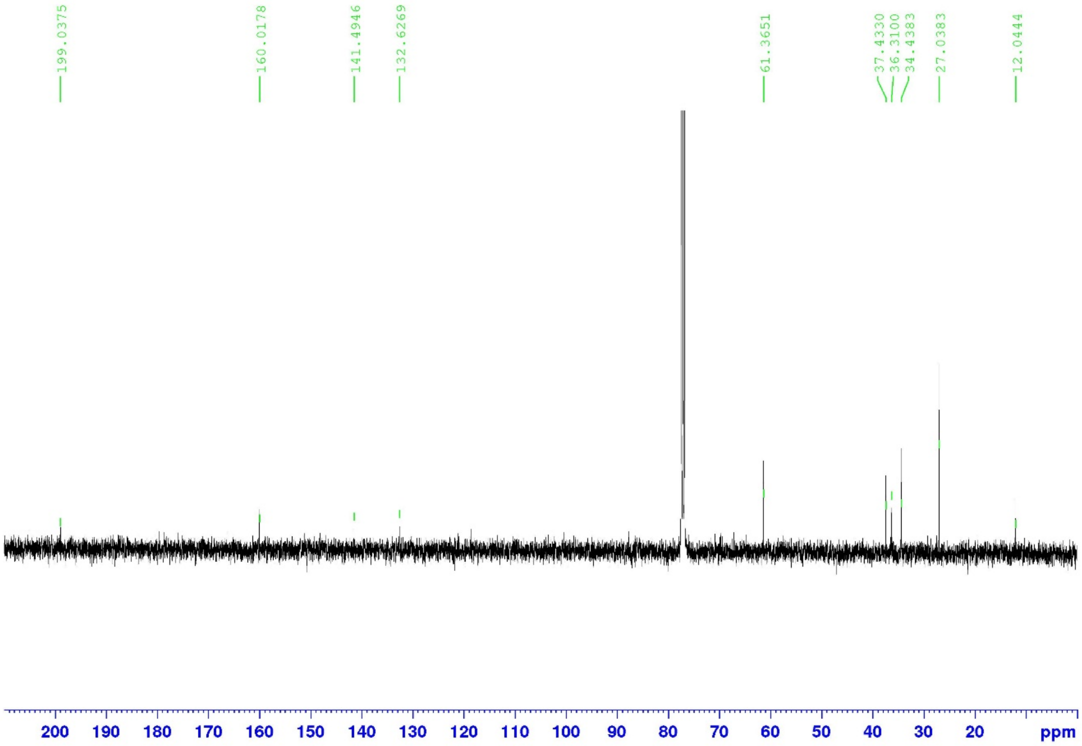

Figure S4. ^{13}C NMR spectrum of compound 3 (3-(2-Hydroxyethyl)-2,4,4-trimethyl-2cyclohexen-1-one) in $CDCl_3$ (125 MHz).

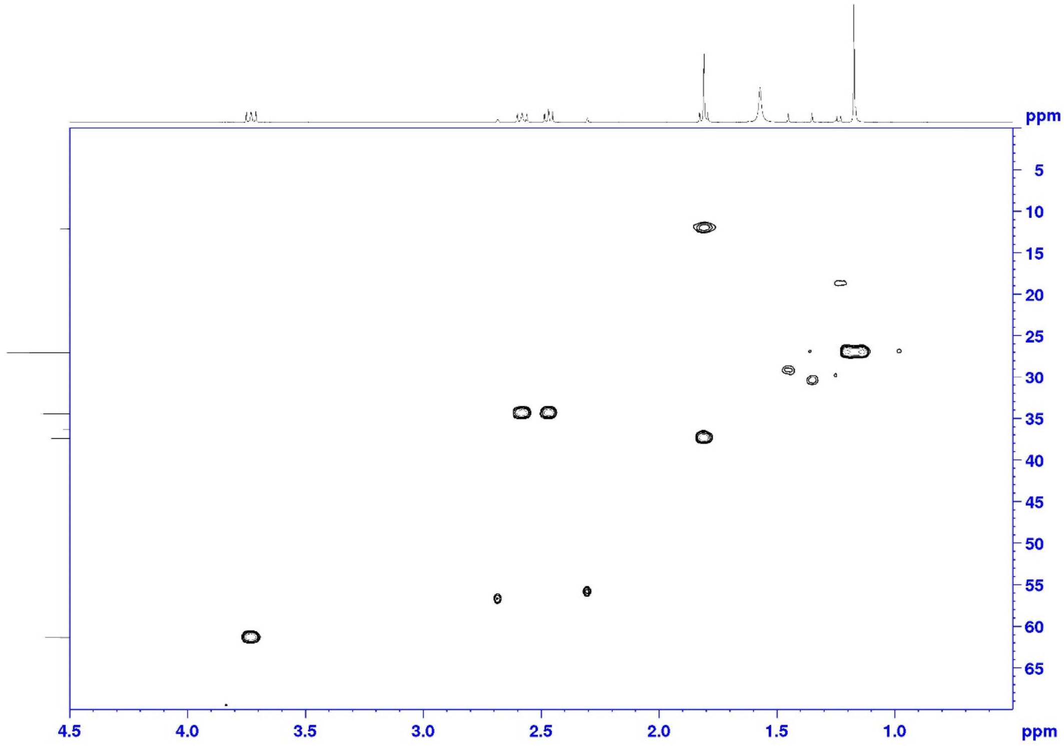

Figure S5. HSQC spectrum of compound 3 (3-(2-Hydroxyethyl)-2,4,4-trimethyl-2cyclohexen-1-one) in $CDCl_3$ (500 MHz).

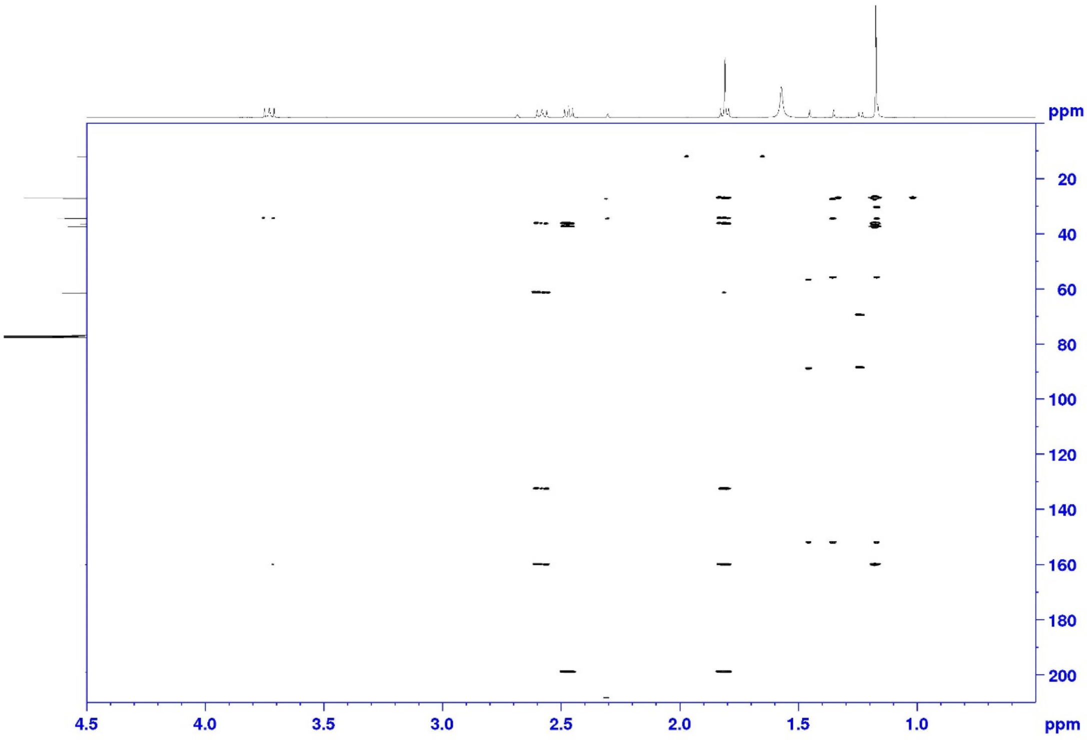

Figure S6. HMBC spectrum of compound 3 (3-(2-Hydroxyethyl)-2,4,4-trimethyl-2cyclohexen-1-one) in $CDCl_3$ (500 MHz).

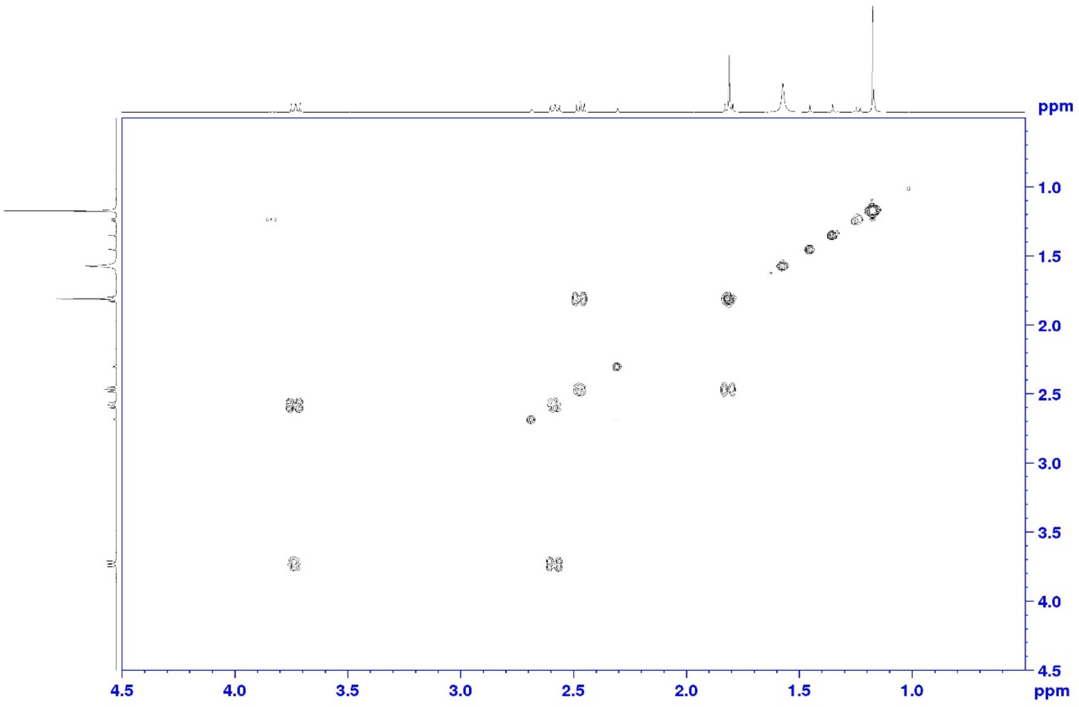

Figure S7. COSY spectrum of compound 3 (3-(2-Hydroxyethyl)-2,4,4-trimethyl-2cyclohexen-1-one) in $CDCl_3$ (500 MHz).

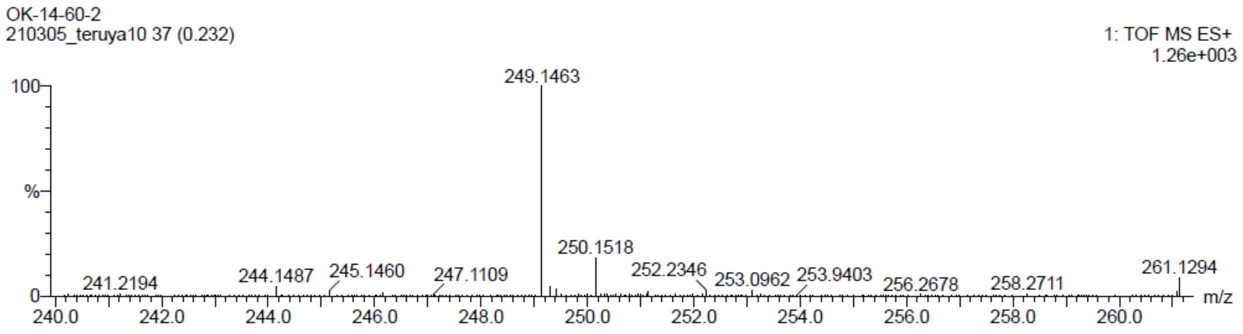

Figure S8. HRESIMS spectrum of compound 1 (4,5-dihydrovomifoliol).

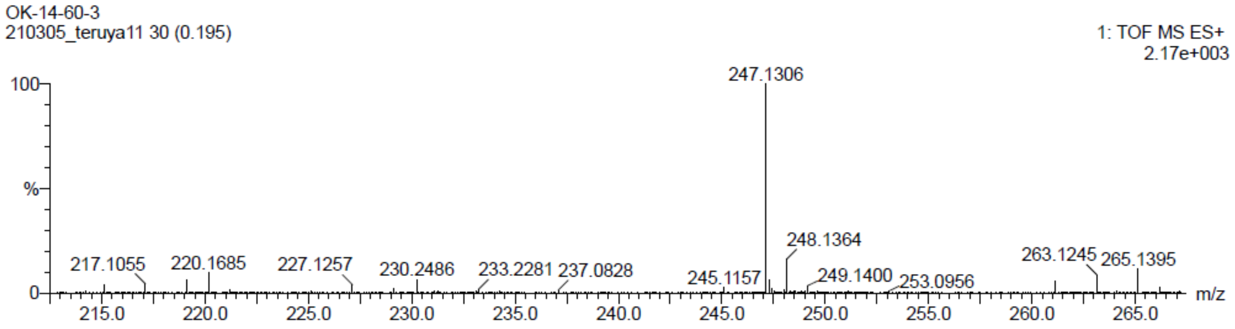

Figure S9. HRESIMS spectrum of compound 2 (3-Hydroxy-5α,6α-epoxy-β-ionone).

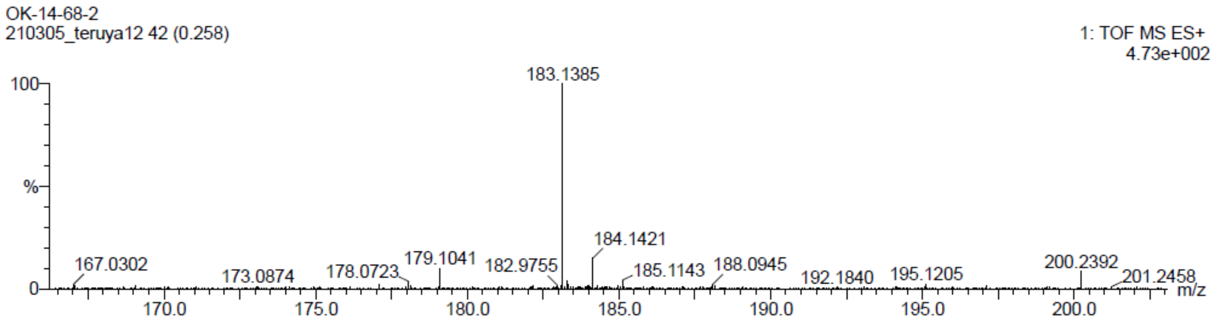

Figure S10. HRESIMS spectrum of compound 3 (3-(2-Hydroxyethyl)-2,4,4-trimethyl-2cyclohexen-1-one).
